# Navigating cognitive strain: a qualitative study on the impact of recent flood disasters on students’ educational experience in Pakistan

**DOI:** 10.1186/s12245-026-01133-0

**Published:** 2026-03-09

**Authors:** Bushra Samman, Caleb Chidozie Chinedu, Sheikh Kamran Abid, Saleh F. A. Khatib, Gerald Guan Gan Goh

**Affiliations:** 1 Faculty of Technical and Vocational Education (FPTV), Universiti Tun Hussein Onn Malaysia, Batu Pahat, 86400 Malaysia; 2 School of Housing, Building and Planning, Universiti Sains Malaysia, Gelugor, Penang 11800 Malaysia; 3 Department of Finance and Accounting, Faculty of Business, Sohar University, Sohar, 311 Oman; 4 Faculty of Business, Multimedia University, Jalan Ayer Keroh Lama, Bukit Beruang, Melaka, 75450 Malaysia

**Keywords:** Cognitive strain, Qualitative study, Flood disasters, Educational resilience

## Abstract

**Background:**

Climate-induced flooding has increasingly disrupted higher education systems, yet limited research has examined how such disasters affect the cognitive and psychological experiences of university students. In Pakistan, recurrent floods have caused prolonged educational interruptions, raising concerns about students’ academic functioning and well-being. This study explores how flood exposure shapes students’ cognitive strain, emotional distress, and coping responses within the higher education context.

**Methods:**

This qualitative study was guided by Cognitive Load Theory and employed in-depth interviews with university students affected by the 2022 and 2025 flood events. Participants were purposively selected based on direct flood exposure and enrollment in higher education during the disaster periods. Data were analyzed using reflexive thematic analysis to identify patterns related to cognitive load, psychological strain, and adaptive strategies.

**Results:**

Findings indicate that disaster exposure intensified both intrinsic and extraneous cognitive load by diverting mental resources away from academic tasks. Students reported heightened psychological distress, manifesting as impaired concentration, memory difficulties, and disrupted study routines. Socio-economic status and geographical location significantly shaped the severity of these challenges. Peer support, community networks, and flexible institutional responses emerged as key buffers that helped mitigate cognitive and emotional strain.

**Conclusion:**

The study demonstrates that climate-induced disasters substantially impair students’ cognitive functioning and educational continuity. By integrating disaster impact scholarship with cognitive theory, this research advances a conceptual framework linking disaster exposure, cognitive load, and educational disruption. The findings offer practical implications for universities and policymakers seeking to strengthen educational resilience and student support systems in climate-vulnerable regions.

## Introduction

Over the past decade, disasters associated with climate change have emerged as one of the most persistent challenges to development and human security worldwide. Floods have increased in both severity and frequency, disrupting the economic and social fabric of affected communities and displacing millions [1, 2]. Recurrent floods in South Asia are now an annual reality, causing massive strain on public infrastructure and recovery systems [3]. Among the most climate-vulnerable nations, Pakistan has consistently been affected by the 2022 monsoon, which affected 33 million people and displaced about 8 million, resulting in estimated damages and economic losses of USD 30 billion [4, 5]. Recently, river overflows and localized flash floods in 2025 have increased these challenges, particularly in peri-urban and rural areas across the Punjab, Sindh, and Khyber Pakhtunkhwa provinces.

Apart from physical infrastructure damage. Disasters are profound social disruptors, affecting everyday life, schooling, work, and mental health of the affected population [6]. Educational institutions are often closed for longer periods due to infrastructure damage, community displacement, and unsafe transportation routes. This commotion results in dropping effects, disrupting examinations, delaying academic calendars, and causing cognitive strain on students, thereby adversely affecting their emotional well-being [7]. The connection between education and disaster has therefore been increasingly recognized as an important area of study within disaster risk reduction and development research. The scholars stress the need to safeguard the continuity of learning during such calamities [8–10].

The theoretical foundation of this research is Cognitive Load Theory (CLT), which is utilised not only as a conceptual framework but also as a prism through which one can view students’ experiences in disaster-impaired situations in the process of gaining knowledge. Using CLT, the paper explores how the demands of cognitive learning are reconfigured by flood-related disturbance and further splinter the intrinsic load by demanding competing academic and survival tasks, the extraneous load by environmental strife and emotional distress, and the germane load by limiting the option to process and combine knowledge effectively. This placement of CLT allows subtle exploration of the functioning of exposure to disasters through cognitive processes in determining educational attendance and persistence among university students in Pakistan.

## Problem statement

Despite growing global recognition of the impact of disasters on education, there is a lack of empirical data on the cognitive demands students face and navigate in higher education. The existing research primarily focuses on infrastructure damage, psychosocial trauma, and school-level attendance [11, 12]. Even less attention has been given to university students, whose academic progress is highly dependent on consistent cognitive engagement when dealing with complex material. University students in Pakistan represent a critical group that is both victims of the disaster disruption and future contributors to national development and recovery [13].

Displacement, financial losses, and commuting challenges related to floods can result in layered cognitive load; the mental effort required to maintain focus, process information, and perform academically. This often exceeds normal learning demands [14, 15]. This load might be compounded by anxiety, logistic hurdles, and economic pressures on students living in flood-affected areas, risking their academic outcomes and psychological resistance [16]. However, empirical research remains scarce on the intersection of disaster impact, cognitive load, and higher education in the Pakistani context.

The lack of qualitative and subjective data on students’ experiences during and after disasters limits policymakers’ and universities’ targeted interventions. Therefore, it is essential to design responsive mental health support and educational policies that can sustain academic success during a crisis.

## Purpose of the study and objectives

The purpose of this study is to examine the influence of recent floods in Pakistan on university student’s educational experiences and cognitive load. This research seeks to uncover the processes of disaster displacement, commuting disruption, emotional stress, and financial strain that shape students’ ability to sustain learning, remain engaged, and meet academic deadlines in their programs. Specifically, the study pursues the following objectives:

1. To examine the affect of flood-related disruption on student’s cognitive processes, including memory, motivation, and concentration.
2. To explore the relationship between disaster-induced stressors and student’s ability to meet academic requirements.
3. To document the coping strategies of students and their adaptive behaviors in maintaining academic continuity in crisis.
4. To create a qualitative intuition that can inform evidence-based interventions and support systems at the university level for disaster-affected students.

Focusing on these objectives can help the study contribute to both the disaster-education literature and cognitive load theory. It will be offering a profound understanding of the intersection between environmental calamity and higher education in a climate-vulnerable country.

## Significance and rationale

This study is significant for multiple reasons, starting with addressing a crucial research gap by focusing on university students rather than the primary or secondary school population, which dominates the disaster-education literature. University students are at an important stage of their educational and professional development; academic interruptions at this time can have a long-lasting impact on careers, graduation timelines, employability, and general socioeconomic mobility. Secondly, incorporating cognitive load theory into disaster research can help advance the conceptual understanding of how environmental stressors intersect with the mental effort required for learning. Existing literature often separates academic disruption from psychosocial trauma; this research explicitly connects them to observe crisis conditions reshaping the cognitive demands of students obtaining higher education.

Thirdly, the findings have practical implications for intervention and policy design. Limited frameworks are regulated by universities and higher education institutions supporting students affected by natural disasters. Generating context-specific, qualitative insights can help this study highlight a strong support mechanism. It will range from trauma-informed pedagogy and flexible academic calendars to counseling services and transport aids, enhancing disaster preparedness across the higher education sector. Lastly, the research contributes to global educational resilience and climate adaptation. Understanding the academic consequences of recurrent floods in a highly vulnerable country like Pakistan, due to climate change that is increasing the likelihood of extreme weather, will provide a valuable lesson for other disaster prone regions.

## Research questions

Grounded in a qualitative study with an exploratory design and guided by the objectives of the research, it is driven by the following central and subsidiary questions:

### Central research question

1. How do university students in Pakistan navigate and experience cognitive load during and after the recent floods, and how does it affect their educational continuity?

### Subsidiary questions

1. What are the ways in which students’ concentration, motivation, and academic performance are interrupted by disaster-related disruptions?
2. How do students manage and perceive the emotional and mental load of continuing their education in flood-affected settings?
3. What support systems or coping strategies do the students use to stay engaged in their studies?
4. What recommendations do the students propose for policymakers and universities to support continued learning during disasters?

These research questions are purposely left open-ended to allow for the subjective experience and narrative understanding of the student’s lived realities, consistent with the reflexive thematic analysis adopted for this study. In conclusion, the research aims to highlight the underexplored areas of disaster exposure, higher education, and cognitive load in Pakistan.

## Literature review

The literature available has consistently shown that flood disasters affect education through structural and logistical means, such as physical infrastructure damage, short- or long-term campus shutdowns, student and faculty displacement, and disruptions to transportation networks. According to global evaluations, floods have become among the most education-disrupting climatic risks, especially in low- and middle-income countries, where institutional recovery capacity is usually low [5]. Education at the tertiary level entails regular attendance, cognitive engagement, and access to learning resources; students’ academic progression and future employability are therefore adversely affected by these factors [17]. South Asian monsoon flooding has been associated with poor attendance, delayed evaluation, and unequal access to educational materials, which are more likely to disadvantage rural and floodplain students [18, 19].

In Pakistan, recurrent floods have become a devastating phenomenon, with the 2022 monsoon floods affecting over 33 million people and causing damage to homes, schools, and transport networks [12]. Post-flood studies have reported that education has been extensively disrupted at both the school and tertiary levels due to the 2022 floods, and the issue has been linked to displacement, destruction, and institutional responses [20]. Floods in 2025 have heightened the vulnerability of both rural and urban communities. The impact of floods has been studied in primary and secondary education [7, 13, 21]. Nonetheless, although the following studies determine the magnitude and structural form of educational disruption, they provide little information regarding the cognitive and psychological experiences of disruption among university students, which is intended to be addressed in the current research. This gap is particularly critical for university students, who are at a developmental stage in their lives and are going through disruption that coincides with their professional preparation, high-stakes assessments, and greater cognitive demands [22]. Table 1 presents studies that analysed the disruption caused by floods among students.

**Table 1:** Existing literature on the impacts of floods on education in Pakistan

Author, Year	Title	Content
Samad and Sheikh [21]	Catastrophic sways of floods on the education sector in Pakistan	Study on flood impacts, damages to school, institutional disruption, administrative shutdown, psychological and economic impacts on learners.
Gull et al. [23]	Assessing educational vulnerabilities in flood-prone areas to identify strategies for promoting education: A case of D.G. Khan district	Focuses on floods affecting learning systems and facilities in a specific district with short- and long-term disruptions
Tunio et al. [24]	Impact of Flood Migration on Education in Flood-Affected Areas of Sindh	Examine the disruption caused by flooding-related migration, including damage to houses, infrastructure, and access to schooling.
Sujaya et al. [13]	Educational Sustainability: An Anthropogenic Study in the Wake of the 2022 Floods in Pakistan	Focuses on educational sustainability after floods, community awareness, institutional resilience, and curriculum adaptation.
Ahmed et al. [7]	The Impact of Flooding on the Education of Children and Adolescents: Evidence from Pakistan	Empirical evidence of long-term and short-term educational outcomes after major flooding in schools
Gul et al. [25]	Impact on girls’ education: District Nowshera, KP, Pakistan	Destruction caused by the 2022 floods in a girls’ school in Nowshera, halting the learning of female students due to school closursse

A systematic review of the literature revealed a lack of studies specifically examining university students with tertiary-level education and the high-stakes assessments and exams delayed by the floods. There are also very few studies focusing on the cognitive and learning losses university students experience during a crisis. There is an emphasis on quantitative studies to measure infrastructure loss, exam performance, and attendance rates, rather than on the lived experiences of students facing cognitive challenges.

There is a pressing need to examine students’ subjective experiences in a qualitative study to understand how floods influence their memory, concentration, and ability to maintain academic engagement in disaster settings. This literature review, therefore, positions the current study to intersect among floods, cognitive load theory, and higher education, proving a conceptual foundation for examining students’ coping strategies and narratives in Pakistan.

### Flood disasters and education disruption

Floods have consistently been the most significant concern to educational continuity worldwide. According to UNESCO (2023), floods accounted for about 40% of total disaster related school closures globally, leaving long-term consequences for learning outcomes, particularly in low- and middle-income countries (LMICs). Wang [26] observed that disasters negatively impact higher education students, as they disrupt academic programs, lead to the loss of social support networks, limit resources, and increase psychological strain. Marin et al. [27] identified the growing need to study the impacts of disasters on education, as there is a significant and adverse relationship between disasters and learning.

Khalid et al. [28] described how LMICs face severe problems compared to high-income countries in the context of educational disruption, compounded by the loss of learning materials, damaged transport networks, and limited economic resources. Aquino et al. [29] raise the question of how populations strongly affected by floods compare with those experiencing fewer consequences due to disparities in resources. Thamtanajit [30] provides empirical evidence of the adverse effects of severe flooding on student achievement, suggesting that additional support can mitigate these effects. Meltzer et al. (2023) specifically conducted research on how university students’ academic and life trajectories are disrupted by flood disasters, acknowledging the unique challenges tertiary students face.

In Pakistan, the 2022 monsoon floods were the most devastating climate event in the country’s history. It destroyed over 26,000 educational institutions and affected millions of learners [31]. Although the reopening of schools is prioritized in national-level recovery efforts, tertiary education remained significantly disrupted, specifically due to roads and bridges being washed out, making it impossible to commute [24]. Ali et al. [32] explored the experiences of school-going children during floods, documenting the psychological effects displacement and loss had on them. However, there is very data on the unique pressures that university students face, including meeting academic deadlines, completing research projects, and often juggling part-time employment [33].

The literature therefore suggests that the educational impacts of floods extend beyond the immediate loss of classrooms and that broader disruptions in students’ lives need to be accounted for to continue learning. The cognitive load increased by the interruption, missed coursework, and managing competing demands post-disaster for university students, underlines the importance of research focusing on their subjective educational experiences and workload.

### Cognitive load theory and educational disturbance

Cognitive Load Theory (CLT) offers a time-tested approach to explaining how learning is conditioned by the capacity of working memory and the allocation of cognitive resources during complex tasks [34, 35]. CLT makes a distinction between intrinsic load (the complexity of learning content), extraneous load (load created by the design of information or learning environments), and germane load (load created by the cognitive resources used to build and strengthen meaningful knowledge schemas). Even though CLT has been extensively employed in instructional design and educational psychology, it is applicable not only to classroom pedagogy but also to situations where external conditions allow a fundamental change in the cognitive demands imposed on learners.

These three types of cognitive loads are redefined differently in disaster-prone environments. The presence of intrinsic load increases because students must cope with academic requirements and immediate survival needs, including finding shelter, caring for displaced relatives, or dealing with financial loss. Learning cognitive complexity does not decrease in the event of a disaster; it is incorporated into an overall set of competing demands that increases mental effort to study academic material.

Meanwhile, the extraneous load also grows due to environmental instability, interrupted learning areas, unstable access to educational material, and increased emotional distress. Such aspects impose cognitive loads unrelated to learning as such and divert attention and working memory from academic processing. Studies show that students in a disaster context face an elevated extraneous load due to factors unrelated to learning itself but competing for cognitive resources [36]. Emotional distress, uncertainty about the future, and anxiety collectively exacerbated the load, reducing the available capacity of working memory for learning [37]. Zhao et al. [38] studied the symptoms of PTSD in students after floods and showed significant worsening over time with reduced academic control and negative emotions.

Disasters also narrow the germane load, which constrains students’ ability to engage in intensive observation to condense knowledge. The fewer resources remain to manage uncertainty, stress, and logistical disruption when cognitive resources are used to manage uncertainty, the fewer resources remain to make meaning and develop a schema. According to stress and cognition research, long-term exposure to uncertainty, emotional strain, and overload negatively affects working memory and executive functioning and, as a result, the transformation of effort into lasting learning performance in learners [39]. In disaster situations, however, cognitive load is not simply augmented but rebalanced, and extraneous and intrinsic demands crowd out germane processes to support the ongoing academic development.

Although it has the potential to explain, CLT has seldom been specifically used in disaster-related educational disruption, especially in higher education settings. The available research is inclined to record learning loss or mental trauma without explaining the mental processes under which the later results are produced [40, 41]. The current research study has developed a conceptual framework to explore the mechanisms of disaster exposure and their impact on student learning, placing the educational disruption posed by floods within a CLT framework. This hypothetical stance establishes the framework for an empirical analysis in which students’ narratives are understood in terms of changing cognitive loads rather than strictly narrow psychological and logistical problems [42].

### Psychological and emotional impact of disaster

The emotional and psychological impacts of disasters have been recognized as central determinants of post-disaster recovery success. Floods have been linked to depression, anxiety, elevated stress, and PTSD symptoms in affected populations [43, 44]. These outcomes end up amplifying due to limited resources, significant losses, and limited institutional support. These psychological burdens can have a profound influence on the emotional well-being, cognitive functioning, and academic persistence of university students, whose lives straddle the domains of emergent academic responsibilities and academic development [8].

Young adults, according to evidence, underscore vulnerability to disaster-related psychological stress due to their developmental stage being marked as a transition in independence, social roles, and identity [45]. Table 2 documents the studies that measured the heightened rates of academic disengagement and anxiety about prospects, further compounding the cognitive strain associated with educational tasks.

**Table 2:** Literature measuring academic disengagement and cognitive load

Author, year	Title	Context
Zhao et al. [38]	Study on the relationship between PTSD and academic control and academic emotion in students after a flood disaster	A longitudinal school study showing PTSD symptoms after flooding predicts reduced academic control. Impaired learning and academic emotions
Mathew et al. [46]	Screening for post-traumatic stress disorder among adolescents following floods, Kerala, India.	Comparative study findings high rates of probable PTSD, concentration problems and sleep disturbance among adolescents after major floods
Wang [26]	Impact of natural disasters on student’s enrollment in higher education programs: A systematic review	Systematic review documents how disasters cause attendance, retention, and enrollment loss and psychological impact for tertiary students
Breen et al. [47]	College students and environmental disasters: A review of the literature	Synthesize evidence on environmental disasters disrupting college students’ mental health, coursework, internships, and life trajectories
Sujaya et al. [13]	Educational sustainability: An anthropogenic study in the wake of the 2022 floods in Pakistan	A qualitative analysis of the 2022 floods in Pakistan found that damaged schools caused psychological stress and disrupted learning pathways.
Almarzouki et al. [40]	The impact of sleep and mental health on working memory and academic performance: A longitudinal study	Longitudinal evidence that poorer mental health and sleep disturbance reduce academic functioning and working memory relevant to the disaster context.
Othman et al. [48]	Flooding: The mental, Social, and Health effects of Flood disasters	A study on students at the University Teknologi MARA discussed mental stress due to flooding
Munsaka and Mustasa [49]	Flooding and its impact on Education	This study explored the impact of disasters on the education system, risking governance failures.

In the Pakistani context, the 2022 and 2025 flood events have increased the concern about students’ mental health. Reports from UNICEF and local NGOs highlight that students are going through a lot due to loss of homes, facing displacement, and prolonged disruption of schooling. There is a noticeable accumulation of cognitive and emotional demands, as students simultaneously prepare for exams or complete coursework whilst dealing with floods. However, this psychological tool was not temporary; it persists in the aftermath of floods, suggesting lingering feelings of uncertainty, fear of recurrence, and survivors’ guilt [50].

Despite this observation, the emotional dimension of disaster exposure and its cognitive and educational outcomes remain underexplored, particularly at higher educational level. There are either researchers talking about psychological distress or academic performance as separate domains, overlooking their interdependence. Therefore, it is essential to present a study that examines emotional well-being as a key factor between disaster exposure and cognitive load, highlighting the importance of holistically addressing students’ lived experiences. This approach will help in designing interventions that not only restore physical access to education but also foster students’ psychological readiness in the aftermath of a crisis.

### Socio-economic and geographic moderators

The impact of floods on education is not evenly distributed; it’s shaped by the intersection of geographic and socio-economic conditions that mediate recovery and exposure.

Students from lower-income families are highly affected by the disaster due to preexisting financial vulnerabilities [29]. Floods typically destroy household savings and livelihoods, reducing the disposable income available for education expenses [30]. For university students in Pakistan with families relying on small businesses, agriculture, and/or informal labor, this can translate into increased financial strain and cognitive burden [32]. Therefore, financial instability increases the likelihood of educational disruption, particularly when combined with heightened emotional stress accompanying disaster recovery.

Geographical location is another moderating factor that measures the severity of the impact. Rural and floodplain communities often face greater infrastructure damage, slower recovery times, and fewer evacuation routes than urban centers with more resilient systems [51]. In Pakistan, rural districts and mountainous regions are particularly vulnerable due to fragile transportation networks that are easily disrupted during flooding [52]. Students from these areas frequently report prolonged absences, interrupted commutes, and delayed re-entry into university life, in contrast to their peers from metropolitan cities [7]. Bigger cities tend to adopt a more hybrid class or emergency scheduling, making it easier for students to cope with this disparity. This highlights the uneven distribution of educational resilience within the country.

Geography and socio-economic status simultaneously interact in ways that increase inequality [53]. Students from affluent families in urban settings have greater access to coping resources, such as a stable Internet connection, housing, or private tutoring. Conversely, students from lower-income households are likely to study in resource-limited environments, thereby intensifying their cognitive load and reducing efficient study time [54].

Despite the significance of moderators, there is limited research examining their combined influence on higher education outcomes in Pakistan. Existing studies tend to treat geographic vulnerability or socio-economic status in isolation, failing to account for the layered vulnerabilities students face in their educational experiences during disasters. This study, therefore, foregrounding these intersecting dynamics, contributes to a nuanced understanding of the segregation between students resuming their studies.

### Coping strategies and support systems

Students’ ability to sustain education in the wake of disaster-related disruption often requires coping strategies and support systems [21]. Coping responses are a typical blend of institutional flexibility, individual initiative, and community-level support.

Individuals frequently adopt problem-focused strategies, such as prioritising essential coursework, reorganising study schedules, and using digital resources to make up for missed classes. Others rely on emotion-focused strategies that include seeking social support, engaging in spiritual or religious practices, or simply taking a break to manage anxiety and stress [55]. These strategies can help mitigate the psychological toll of disaster; however, their effectiveness varies widely depending on access to stable Internet connectivity, housing, and quiet spaces to learn.

**Fig. 1:**
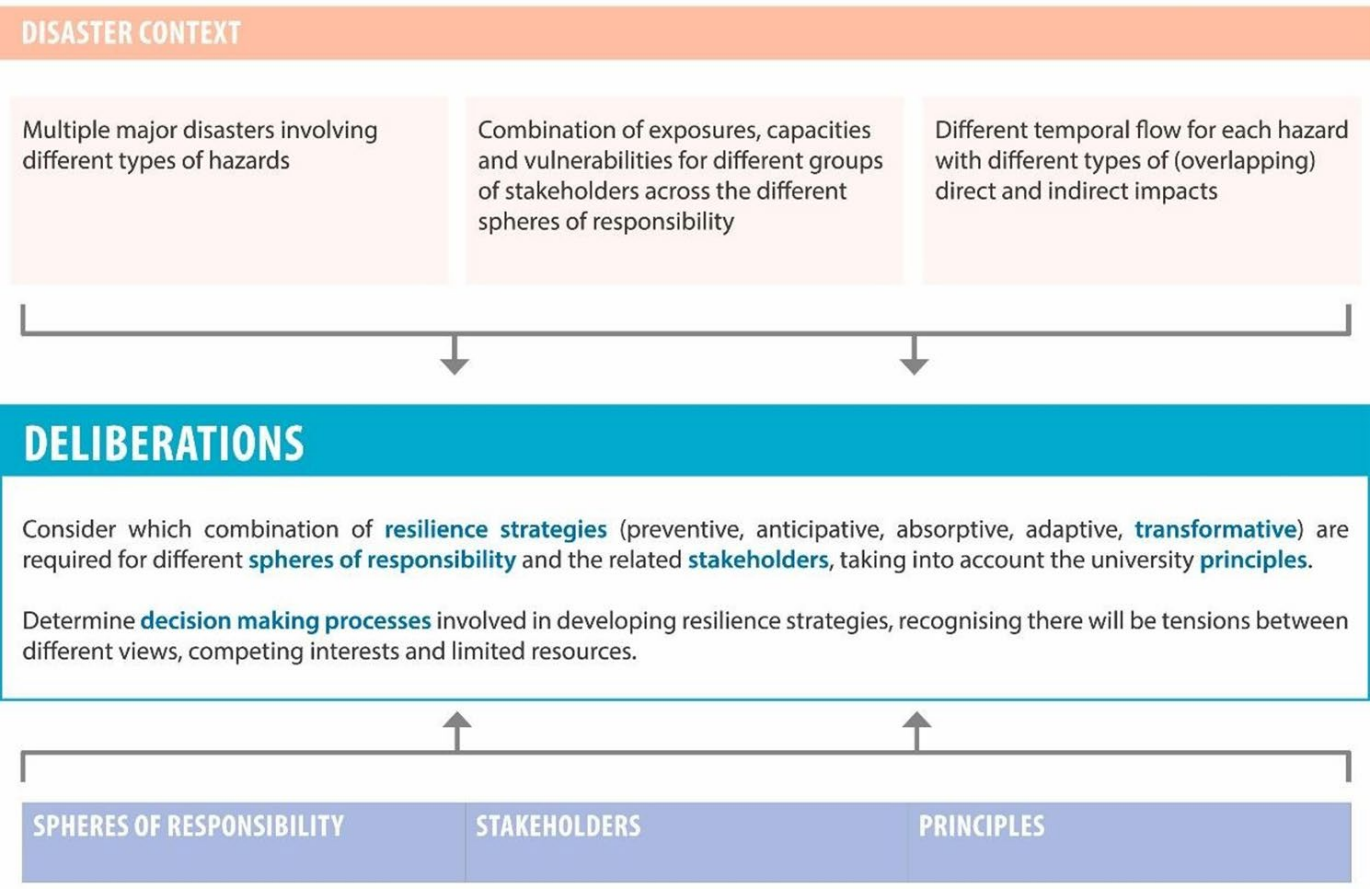
Framework for disaster resilient universities. Source. [33]

Institutional support plays a significant role in shaping the post-disaster educational continuity. Institutes offering remote learning options, flexible deadlines, and counseling services are better positioned to help students cope with the cumulative cognitive load of disaster recovery [21]. Research from existing flood-affected regions has shown that students exhibit lower dropout intentions and greater satisfaction when their universities actively communicate contingency plans and provide psychosocial support [27]. In Pakistan, such institutional responses have been uneven. Urban universities have adopted online platforms and provided counseling, whereas smaller universities have insufficient mental health resources and bureaucratic rigidity, making it hard for them. Figure 1 shows the framework laid by Gibbs [33] for disaster-resilient universities.

Apart from universities, NGO’s and community interventions also contribute to student resilience. The initiatives of temporary learning centers, textbook and digital device distribution, and the provision of subsidies have been documented to help address educational interruptions during disasters [54, 56]. In 2022, during the floods, several NGOs coordinated with local authorities to arrange emergency stipends and safe commuting routes to help students on lower incomes. These efforts have addressed logistical barriers and conveyed a sense of social solidarity, thereby stabilizing psychological effects [56].

Despite these efforts, qualitative accounts from students have often found the support system to be delayed, insufficiently targeted, and fragmented in addressing their specific needs [18, 19]. This points to a gap in the current understanding of critical recovery from disasters, limiting insight into how students experience and evaluate their mechanisms in the context of emotional and cognitive strain. This present study seeks to capture students lived experiences, shedding light on the familial, institutional, and community resources needed to maintain academic momentum in the aftermath of floods.

### Theoretical integration and conceptual framework

Cognitive load theory and the coping literature provide a comprehensive perspective on how floods shape students’ educational experiences. Disasters disrupt the material aspects of learning while imposing significant psychological and cognitive demands. CLT therefore offers a valuable framework for conceptualising these effects. Intrinsic load intensifies as students try to balance survival-related concerns with academic responsibilities. Extraneous load rises due to displacement, instability, and emotional distress, and germane Load minimises as cognitive resources are redirected away from deeper learning processes, such as meaning-making [34, 57].

**Fig. 2:**
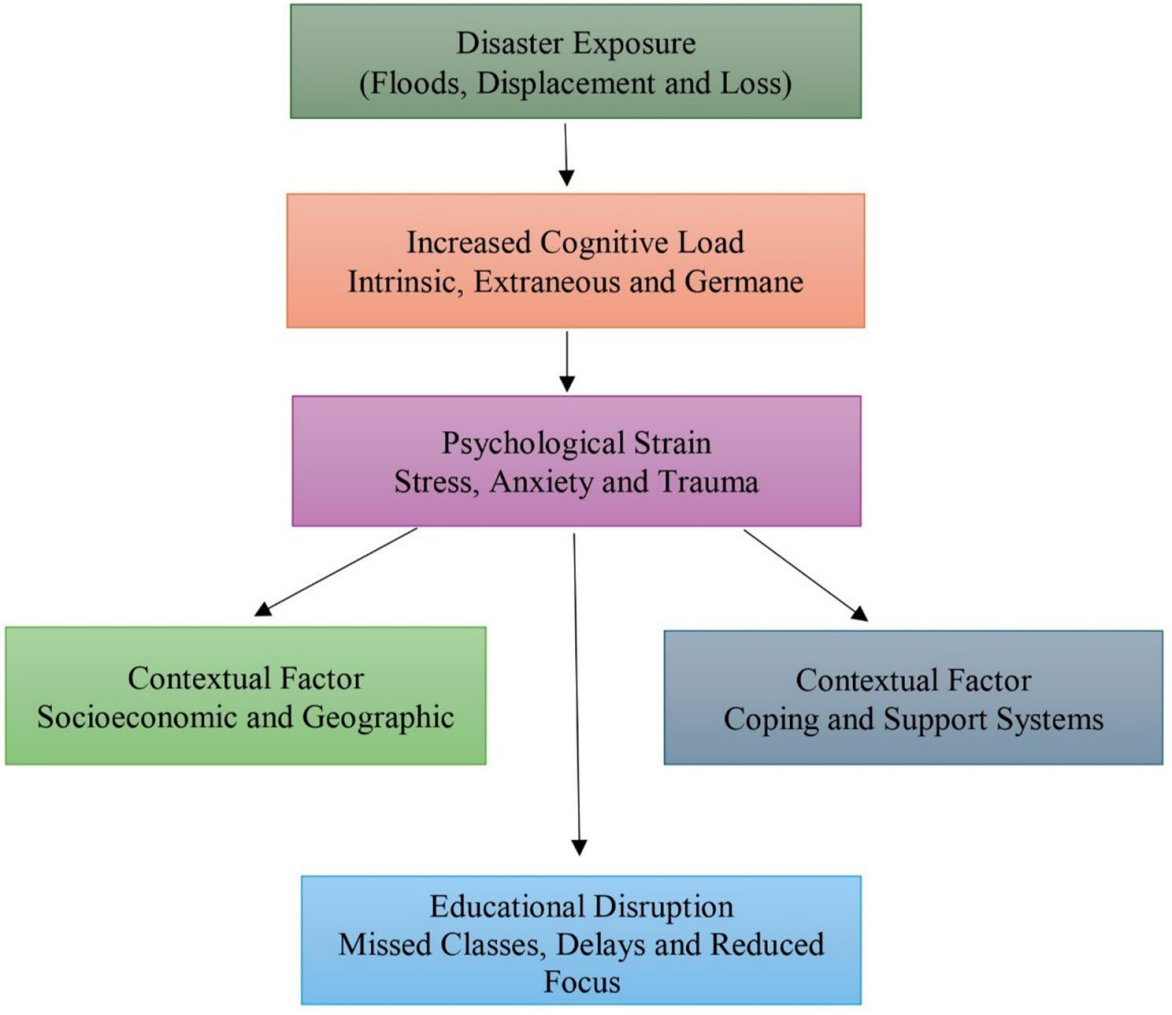
Theoretical integration

This framework underscores disaster recovery as a logistical process that fundamentally alters cognitive efficiency and economic outcomes. Figure 2 shows the emergent framework illustrating how disaster exposure shapes psychological strain, cognitive load, and educational disruption, with influences from socio-economic and coping factors.

Introducing CLT into disaster impact studies will further emphasize the role of psychological well-being and the effects of socio-economic and geographical contexts on it [58]. Vulnerable students often face prolonged disruptions and fewer recovery opportunities; therefore, coping mechanisms and institutional support are central. Flexible academic policies, counselling interventions, and peer networks within the community can help alleviate the extraneous load, enabling students to redirect their attention to educational tasks.

This conceptual framework outlines visions of disaster exposure that trigger layered emotional, cognitive, and structural challenges, shaped by contextual and personal factors. A qualitative approach is therefore well-suited to unravel these dynamics, offering insight into how students perceive and navigate intersecting pressures. It also contributes to theoretical advancement and informs interventions to promote academic resilience in flood-prone regions.

### Research gap and justification for the current study

Although there is growing scholarly interest in the educational implications of climate-related disasters, several gaps remain. To begin with, much of the available literature mainly deals with primary and secondary education, and very little has been done regarding higher education students. The role of university students in the context of disasters has a specific location: they face complex academic tasks, life transitions, and, in most instances, the need to earn money and often to support their families. Nevertheless, their experiences with education in disasters and post-disaster are not well represented, especially in flood-infested countries like Pakistan.

Second, although there is quantitative research on the relationships between disaster exposure and educational status, including attendance disruption, retarded progression, and academic scores, the field of qualitative, narrative-based research that explains how students personally perceive and cope with the disruption is markedly deficient. Quantitative indicators have useful scope but little information about the cognitive and situational processes in which the educational challenges are played out. In Pakistani circumstances, where recent floods have led to long-term, unequal disruption of regions, a balanced understanding of the resilience and vulnerability in education is limited by the dearth of student voices.

Third, previous studies in disaster education research have seldom used CLT as a method of interpretation. Though stress, psychological strain, or loss of learning may also be mentioned, rarely are studies that explicitly examine how the conditions of disaster reorganise cognitive demands on learning or distinguish between intrinsic, extraneous, and germane cognitive load. This theoretical underapplication restricts the field’s capacity to discuss how and why educational disruption occurs at the cognitive level, especially in higher education settings.

It is in these areas that the current research will adopt a qualitative design to investigate the educational experiences of Pakistani students at the university in the wake of recent flood disasters, using CLT as an analytical tool. The research directly relates to the research questions that aim to comprehend the role of exposure to a disaster in recreating cognitive demands, breaking down learning processes, and shaping students’ opinions about the presence of institutional support in higher education, by foregrounding students’ narratives.

## Methodology

### Research design

This study adopts a design situated within the constructivist-interpretivist paradigm and uses qualitative research methods. The research aims to explore how students’ cognitive load and educational experiences are shaped during the current floods in Pakistan. Rather than imposing predefined categories, it is essential to attend to students lived realities; therefore, a constructivist epistemology is appropriate. The reflexive engagement between the participant and the researcher allows the study to show the complex interaction among disaster, education, and cognition within a socially and geographically specific context.

The research design used in this paper is an exploratory, quantitative approach that employs methods to yield context-specific understandings rather than statistically generalizable results. The design is strategically located within the social, geographic, and institutional realities of flood-prone areas in Pakistan, as students’ educational experiences are locally grounded in disaster, infrastructural, and socioeconomic dynamics. The study aims to shed light on the experience and interpretation of cognitive and educational challenges in this environment by anticipating participants’ narratives and taking care not to generalise the argument beyond the scope of the sample, which is a qualitative study.

### Participants and sampling

The participants in the study were purposely selected to reflect the maximum variation across gender, academic level, geography, and severity of flood exposure. There were 12 university students across Pakistan, meeting the following inclusion criteria: (a) they were enrolled in a university program during the 2022 and/or 2025 floods, (b) their education was directly affected by the said calamity, and (c) they experienced either loss of livelihood, direct displacement, or significant commuting disruption.

Participants were recruited using a criterion-based, purposive sampling strategy. University contacts, local NGOs, and student unions involved in flood relief were approached to identify eligible students. To avoid underrepresentation, remote areas and female participants from conservative households were reached by snowball sampling. Recruitment materials emphasized confidentiality and voluntary participation. Participants were provided with referral information for psychosocial support if needed.

The diversity of participants ensured coverage of multiple educational disruptions across northern mountainous districts, floodplain regions, and urban centers. Damaged roads and bridge closures, missed labs, delayed thesis work, and deferred exams were all observed. Students’ socio-economic backgrounds vary from rural agricultural households with massive loss of property and wealth to urban middle-class families facing psychological strain despite minimal material loss.

Table 3 reflects the heterogeneous exposure across provinces to represent students from multiple geographical areas. This diversity enriched the data, enabling analytical comparisons among students with varying levels of exposure to floods, educational disruption, and displacement duration.

**Table 3:** Participant demographics

Participant	Age	Gender	University	Field and year	Residence
P01	19	F	Univ. of Peshawar	BS Biology, 1st Year	Buner, KPK
P02	22	M	Univ. of Poonch	BA English, 3rd Year	Rawalakot, AJK
P03	20	F	Univ. of Punjab	BSc Comp. Sci., 2nd Year	Southern Punjab
P04	23	M	Univ. of Karachi	BBA, Final Year	Thatta, Sindh
P05	21	F	Sindh University	BEd. 2nd Year	Interior Sindh
P06	24	M	BZU Multan	MSc Physics, 1st Year	Multan, Punjab
P07	18	F	Univ. of AJK	BSc Nursing, 1st year	Muzaffarabad, AJK
P08	25	M	Univ. of Swat	MA Sociology, Final Year	Sawat Valley, AJK
P09	20	F	Univ. of GB	BS Env. Sci., 2nd Year	Gilgit
P10	22	M	GCU Faisalabad	BSc Engineering, 3rd Year	Faisalabad
P11	23	F	KIET Karachi	BBA, Final Year	Hyderabad, Sindh
P12	21	M	Univ. of Peshawar	BA Pol. Sci., 2nd Year	Charsadda, KP

### Data collection

The data were collected using semi-structured in-depth interviews following an outlined protocol. Interviews were conducted via secure online platforms. Each interview lasted 30–45 min and was conducted in the participants’ preferred language (Urdu and/or English) to ensure no context was missed. Field memo captured nonverbal cues, environmental disruptions, and researchers’ personal reflections after each interview.

### Data analysis

Data were analyzed using Braun and Clarke’s [59, 60] Reflexive Thematic Analysis framework. The researcher, who was first familiarized with the transcripts, repeatedly read them and took notes. Coding when data-driven and inducted with codes applied line by line to capture the cognitive load indicators, educational challenges, and emotional responses. Codes were combined into candidate themes, which were then analyzed iteratively, reviewed, and refined to ensure consistency across the dataset. Themes were generated using both cross case synthesis and within-case depth to analyze multiple students’ shared experiences and one participant’s subjective story, respectively. The analysis was conducted manually, without software, to maintain an analytical interpretation as human-centred and reflexive as possible. *Reflexivity and Analytic Decision-Making*.

The analysis was conducted using a 6 step reflexive thematic analysis method, guided by the principles outlined by Braun and Clarke [59, 61]. Themes in this method are not objectively discovered in the data but are interpretive constructions co-created through a lengthy process of researchers interacting with participants’ accounts. It was thus an active, reflexive process of meaning-making via repetitive reading, coding, and refining themes, and is not the mechanical application of pre-established categories.

The first stage of coding was inductive, focusing on what participants said about the educational disruption, cognitive strain, emotional reactions, and coping behaviours. Here, data-driven codes were deliberately chosen and not constrained by the CLT, allowing patterns to be observed based on how participants themselves described their experiences. Later in the analysis, the CLT was proposed as an analytical prism for subsequent stages, shaping the process of refining themes and organising ideas conceptually. Namely, CLT was applied to investigate how participants’ narratives revealed changes in intrinsic, extraneous, and germane cognitive load during disaster circumstances, without imposing the data on existing theoretical frameworks.

During the analytical process, the researchers engaged in reflexive memoing, writing down interpretive choices, emerging assumptions, and changing theoretical understandings. The process of memoing facilitated transparency by documenting how some codes were merged, redefined, or shelved, and how the analytic attention shifted as more attention was focused on the data. Themes were narrowed to a coherent, consistent comparison across transcripts, and themes were also separated.

This process was made possible by researcher reflexivity. The scholars were conscious of their role as scholars operating in the contexts of teaching and learning, where disciplinary backgrounds, prior experience in disaster and education studies, and perceptions of student vulnerability might affect interpretation. Reflexive practice entailed challenging how specific narratives were framed, the application of theoretical concepts, and the use of analytic decisions to construct findings. Such a reflexive orientation also makes the analysis a more credible piece of work, as it helps see the interpretive work through which the themes were constructed.

### Trustworthiness and reflexivity

The credibility of the study was enhanced through peer debriefing to discuss each coding frame, emerging interpretations, and potential biases. A reflexive journal was documented and maintained to show researchers’ positionality and analytic decision-making. Member checking was also selectively applied to share brief thematic summaries with five participants, who confirmed that their interpretations were represented as intended and resonated with their subjective experiences.

### Ethical consideration

Confidentiality was maintained by anonymising all transcripts and assigning each participant a pseudonym. In every interview, it was made clear that participation was voluntary, and all participants were reminded of their right to discontinue if any inconvenience occurred. Since the study talked about psychological struggles and disruption, sensitivity was taken in order to avoid any emotional triggers, and the participants were offered support if needed.

This study was conducted as qualitative, survey-based research. The project focused on flood disaster and disruption. It did not involve experimentation, clinical procedures, or the collection of biomedical data. Under research regulations, such studies do not require approval from a medical or bioethics committee. However, the research was conducted in accordance with institutional ethical guidelines and adhered to the ethical principles outlined in the Declaration of Helsinki. These considerations ensured that participants’ rights and well-being were safeguarded and that the study maintained the highest standards of integrity.

## Findings

### Theme 1. Cognitive strain and emotional impact

This theme involves subjective experiences of cognitive load and emotional pain of students immediately after the floods and how the subjective perspectives of worrying, fearing, and feeling emotionally drained influenced their capacity to stay mentally focused in educational institutions. The interviews revealed that the most profound and immediate impact recent floods had on students was the intense cognitive and emotional strain they caused. Nearly all participants described having gone through excessive mental load that lasted long even after the floods.

*I was physically present in the class*,* but my mind was stuck with my family*,* whether they are safe or not*,* what happened to our house (P02).*

*Sometimes I just stared at the board without understanding anything. It felt like my brain was fully occupied with all the wrong things*,* fear*,* worry*,* stress (P06)*.

This cognitive strain was sensed in the compound as emotional fatigue; participants described feelings of sadness, demotivation, and helplessness, leading them to feel that studying was secondary and burdensome. P07 summarized this feeling well:

*Even after the flood was over*,* I felt drained and couldn’t push myself to do the schoolwork. It felt like the water took my energy with it.*

The statement mentioned above resonated across multiple interviews, with nine out of twelve participants using the term, “numb”, “exhausted”, “burned out”. Emotional fatigue appeared to have created a feedback loop, poor academic performance triggered the stress and anxiety further, deepening the fatigue.

*I kept checking the weather updates*,* it’s hard to focus on knowing this could happen anytime. The rain now makes me anxious and panic even if it’s small (P05).*

This anticipatory anxiety was highlighted as a psychological impact of disaster that extended beyond the recovery period. Many students considered education as a backdrop of ongoing uncertainty and hypervigilance. It shapes their ability to learn, remain engaged, and concentrate on their education.

### Theme 2. Disruption of the learning environments

This theme investigates how disruption of physical, institutional, and domestic learning environments caused by flooding affected students’ educational patterns and study conditions. Another recurrent theme across the interviews was the logistical and physical disruption to students’ learning environments. Most participants faced university closures after the floods, which lasted from one to three weeks, disrupting their academic schedules.

*Our university was closed for almost three weeks and by the time we returned we had lost almost half the semester’s pace, we had to rush through many lectures and projects, it made the process more stressful, and this being our final year did not help (P10)* Several participants noticed that the physical damage to the libraries, classrooms, and computer labs persisted, and that learning spaces were not fully functional even after the university opened.

*The computer lab at our campus was flooded. When we went back*,* half the systems weren’t working*,* so we had to take turns using them. This delayed our assignments and work further (P4).*

Transport disruption was also noted as a key barrier for students living far away from the campus. Lack of public transport, damaged roads, and longer travel times have made it difficult for them to attend regularly.

*I missed several classes even after the university reopened because buses and rickshaws weren’t running. Even if I could go*,* it would take double the time to reach*,* and the whole commute would tire me out before I got there (P06).*

The disruption was not only limited to infrastructure but also the environment at home. Many participants reported that the shared spaces they were in were so crowded and loud during recovery.

*Our house was full of relatives who had evacuated their homes. It was hard to find a quiet corner to study. I tried to study at night*,* but there was also someone in need of help or just too much noise (P08).*

This theme demonstrates that the disruption affected not only universities but also homes, creating multiple levels of difficulty for students. Even for students with access to online materials, unreliable electricity and unstable internet made remote studying an impossible task. *We tried group discussions on WhatsApp*,* but the electricity went out for almost all evenings. Sometimes the phone would die*,* and I couldn’t charge it till morning (P05)* The combination of damaged infrastructure, school closures, transport issues, and unsuitable study conditions at home collectively adversely impacted students’ ability to study [62].

### Theme 3. Academic performance and motivation

This theme examines the impact of chronic disruption on academic performance, motivation, and future shifts, as students struggle to sustain progress amid repeatedly constrained and unstable academic schedules. Another key finding was a significant decline in students’ motivation and academic performance during and after the floods. Nearly all participants reported missing deadlines, falling behind in their coursework, and postponing exams, which caused frustration and stress. This interruption to the academic calendar forced students to compress their workload into a shorter timeframe, leading to decreased comprehension and rushed learning.

*I missed two internal tests because of the floods and when I went back the teachers just gave us extra assignments instead of the makeup tests. It felt like I never learnt that material (P07).*

*When university reopened*,* we had double the amount of work. The pressure to catch up was immense. Teachers were rushing the syllabus*,* and we didn’t get proper time to understand anything (P11).*

Motivation to study was also impacted as students found it hard to concentrate even when they had time. They also mentioned that academic instability led them to question their long-term goals [63].

*I would open the books and then just stare at the pages blankly. My mind was somewhere else*,* thinking about whether we should repair our house and if so*,* how much money we are left with and such thoughts (P09).*

*I started to wonder if it was worth continuing the study*,* if disasters were to keep interrupting every year what’s the point? What if it’s the same next year?*

However, not all responses were negative; some participants shared that the floods had made them determined to succeed academically.

*It made me focused on completing my degree. I felt like if I could get through this hard phase of my life and still graduate on time I would be able to face anything in life.*

These findings suggest that, even though most students experience a decline in performance and motivation, a small subset develops stronger academic resilience, using disasters as a motivator to work harder. This aligns with the existing post-traumatic growth literature, where adversity fuels determination and renewed purpose. Overall, this theme centered on the academic disruptions during disasters as not only logistical but also psychological hardships for students, impacting their competence, confidence, and future aspirations.

### Theme 4. Emotional strain and cognitive load

This theme builds on the previous ones, emphasising the role of emotional strain as cognitive load and demonstrating the processes by which flood-related stress overloaded students’ working memory and disrupted learning. One of the most frequent findings across the interviews is the extent to which the floods and their aftermath occupied the students’ minds. Students were not only physically disrupted and displaced but also were mentally occupied which had significant adverse impact on their ability to learn and retain information.

*Even after we returned home*,* my brain felt very heavy. I would just sit with my books*,* but I could not absorb anything. It was like my mind was still back in the moment*,* watching the water rise and worrying if it would come back again (P06)*.

This sense of mental saturation was increased when multiple simultaneous stressors like financial concerns, loss of property and family obligations, came together. Participant P09, while describing his typical day, mentioned that after helping clean the house and checking all the necessary items, when he sat down to study, his head was already filled with electricity, repairs, and money, making it harder to focus.

This cognitive load was about practical responsibility and emotional processing. Students mentioned their fear of recurrence every time it rained which acted as a constant background distraction

*When the sky turned dark*,* my heart would race. I would grab my phone and check the news about more rain warnings instead of studying. Even in class*,* if I heard heavy rain outside*,* I would lose track of what the teacher was saying and completely lose my focus*.

#### (P10)

For some students, this translates into memory lapses and difficulty organizing their work.

*I forgot deadlines I had written down in my diary. I even submitted one assignment twice because I was so confused. It wasn’t laziness*,* it was like my mind was scattered everywhere (P12)*

There were also physical symptoms of this emotional burden; participants spoke about irregular eating patterns, poor sleep and headaches. This, together, contributed to the reduced cognitive capacity they were facing.

*Nights were noisier because everyone in the neighborhood was awake*,* trying to pump water out of their houses. I slept for maybe 3 h a night. The next day in class I could barely keep my eyes open. I had a hard time remembering what I had studied (P08).*

This team also highlights the personal and academic stress, reinforcing each other. Like a loop, if students fell behind in their studies, they became more anxious, which further made it hard for them to concentrate, creating a vicious cycle. However, participants also reported that they eventually developed coping mechanisms to manage this cognitive strain.

*I kept thinking about how much I was missing. Then when I sat to study*,* I was too anxious to do it. It was like my brain refused to cooperate with me (P07) I started writing everything down*,* our To-Do List for the house*,* a To-Do List for my classes. So basically*,* I was trying to not keep anything inside my head*,* and it helped reduce my stress a little and have a better focus (P11)*.

Collectively, the narrative of theme 4 paints a clear picture of floods imposing a dual load (practical and psychological) on students that exceeds their cognitive capacity. It aligns with cognitive load theory, which states that working memory can become overloaded, impairing the ability to process and learn new information. This study shows that the students were not just distracted but also mentally overstretched. Addressing cognitive stress through counselling, structured study support, and phased return-to-class schedules could significantly improve student outcomes in disaster-affected areas.

### Theme 5. Coping mechanisms and resilience

Although widespread disturbance was experienced, this theme underscores how students coped and adapted their practices to address cognitive pressure and continue their academic activities during and after the floods. Despite the emotional and cognitive burdens, they faced, the students also showed remarkable resourcefulness in finding ways to maintain a sense of normalcy and continue their learning [64]. This theme highlights the strategies students used to cope with this disruption. It ranges from informal peer study groups and schedule adjustments to emotional resilience and future-oriented motivation. Many students turned to peer networks as a primary source of emotional and academic support.

*We started meeting at one friend’s house every evening because her home had electricity before ours did. We would share notes*,* do assignments together and even cook simple meals so we wouldn’t waste time running back and forth. It made studying easier and less lonely (P07)*.

*Whenever one of us felt discouraged or anxious*,* the others would cheer her up. We even shared jokes about studying by candlelight*,* it helped us laugh at the situation and not feel hopeless (P10)*.

This collaborative approach helped them reduce the economic burden and also provided the emotional solidarity they needed. Students mentioned that simply being around others who were facing the same struggles made their situation a little more bearable. Support from supervisors and teachers also came in various degrees, one participant mentioned a professor personally calling her students to check on their situations.

*She called telling us not to panic and that the deadlines would be extended. That called meant so much to me because it felt like the university cared about us. It motivated me to keep going rather than give up (P12).*

Some participants describe practical strategies to manage their cognitive load and time. P11 found that making return schedules and breaking tasks into smaller chunks gave her a sense of control. Others turned to technology where possible, using mobile data to access lecture notes or for video calls for group discussion. However, this was often interrupted by poor connectivity. Interestingly, several participants highlighted spirituality and religion as sources of strength.

*After the floods I started praying more regularly. It calmed me and reminded me that it was a test, not the end of everything. It gave me patience to keep trying (P09)* For some people, a crisis became a motivational turning point, prompting them to commit to education long term. P08 mentioned that he realised the fragility of life and that education would not wash away in floods.

This theme revealed that resilience was not just about bouncing back but also actively adapting and innovating within extreme situations. Students found ways to shape their learning opportunities in response to the adversity they faced, whether by developing better time management habits, rethinking their career goals, or forming stronger social bonds.

At the same time, participants emphasized that resilience should not mean leaving students to cope entirely on their own. They called for systematic institutional and policy-level support to complement their efforts.

*We can support each other to a point. We also need universities and governments to have proper plans. Otherwise*,* we’re not learning anything*,* just surviving (P10)* Theme 5, therefore, concludes the narrative by showing that, while the floods created immense disruption, the students were not passive victims. They engaged in coping mechanisms, demonstrating perseverance, solidarity, and creativity. Yet that experience underscores that resilience should not be romanticized; structural interventions should support it to ensure an equitable economic recovery.

Besides personal and peer-level coping mechanisms, the participants also considered what kinds of institutional support they felt they needed to maintain their presence in the educational process during the recovery of the disaster. Students explained their expectations as focused on flexibility, communication, and apparent institutional concern rather than official policy change. Several respondents reported that long deadlines, instructor support, and recognition of disaster-related limitations minimised their feelings of academic pressure.

These comments were based on personal experience, and students highlighted how the level of institutional responsiveness, or lack thereof, affected their ability to manage academic stress when their cognitive and emotional load was greater than usual. *We can help one another up to a certain extent. Universities and governments should also have the right plans. Otherwise*,* we are not learning anything*,* we are just surviving (P10)*.

## Discussion

The results of the present research can be logically explained within the framework of the CLT, which presents a systematic approach to understanding how disaster-related disruption alters students’ learning processes. Across the themes, participants’ narratives indicate a reorientation of intrinsic, extraneous, and germane cognitive load rather than an increase in academic difficulty. The combination of intrinsic loads stemming from coursework, displacement, family issues, and financial loss amplifies the inherent load, as the intrinsic complexity of academic work is forced into a clash of demands to survive.

At the same time, interrupted learning conditions, insecure transportation, ambiguous institutional communication, and constant emotional distress all act as sources of extraneous load, that imposes mental strains that are not related to the actual learning. These states inhibit germane load, restricting students’ capacity to engage in prolonged generation of meaning, reflection, and synthesis of academic knowledge. Interpreted thus, the evidence shows that exposure to floods disrupts the educational process via cognitive processes, and this can be extended to disaster-affected higher education settings with CLT as well.

Students experienced heightened cognitive overload during and after the floods, according to the study’s findings, often reporting difficulty remembering lessons, staying motivated, and concentrating to engage in schoolwork. This aligns with Sweller’s CLT, which states that external demands hinder learning and impair working memory [58]. However, according to our participants, cognitive strain was due to academic tasks, with disaster-related stressors amplifying it. Displacement, uncertainty about future schooling, and infrastructure were among the most common stressors among participants, extending previous studies on disaster-related educational disruption [48]. The literature demonstrates that psychological strain directly affects the learning process. Therefore, the findings underscore the need for post-disaster interventions that address both academic and psychosocial dimensions to optimise student recovery outcomes [49].

Participants consistently mentioned having difficulty focusing on the academic work due to heightened feelings of helplessness, anxiety, and sadness following the floods. This finding links disaster exposure to increased psychological stress among students, as discussed in previous literature [36, 46, 65]. Our participants emphasised the prolonged period of reduced emotional and cognitive capacity for learning, as the stress persisted well beyond the immediate aftermath of the disaster. Emotional strain causes transient responses and sustains barriers to educational engagement, extending the research on disaster mental health. School recovery plans are essential for integrating mental health supports, including peer support programs and counselling services, to mitigate the long-term impact of disasters on students.

Even at the level of structural factors leading to cognitive outcomes, disrupted learning conditions in the form of unstable access to campus facilities, interruptions in transportation, and lack of institutional communication can further explain how such conditions can be transformed into cognitive outcomes. The conditions do not modify the academic content but add an extra load to get through it, which supports the idea of CLT’s distinction between intrinsic and extraneous load. Notably, the results indicate that when extraneous load is unrelenting, students’ capacity to invest cognitive resources in germane processing, such as deep learning and knowledge integration, is impaired. This understanding contributes to the study of disaster education because it shows how cognitive processes lead to educational disruption rather than solely logistical or psychological processes.

The interviews highlighted various coping strategies that students applied to manage stress and restore routine. Some participants relied on collective support from teachers, peers, and families, while others relied on resilience, emphasizing self-discipline and perseverance [66]. Several students described study groups in shelters as the only way they could keep up with schoolwork amidst the chaos. Echoing previous studies highlighting peer networks as protective against trauma, underscoring the critical role of social connectedness in a disaster. Participants also mentioned the vital role of spiritual and religious gatherings, suggesting that coping is multidimensional. Instilling the necessity of disaster recovery interventions going beyond material aid. Culturally relevant and community-driven approaches can support students’ social and psychological well-being.

Participants repeatedly expressed frustration with slow responses from institutions and the lack of structural preparedness within educational systems. Students mentioned being forced to study in overcrowded classrooms and makeshift spaces because parts of the school were left unrepaired for weeks and even months. The cognitive load was a byproduct of personal stress, intensified by systemic inefficiency and infrastructural neglect. This resonated with the broader literature on post-disaster education, emphasising institutional readiness and timely interventions to minimise learning loss [30, 31, 41, 46, 67]. Psychosocial support programs are absent in most institutes, raising recurring concerns and suggesting a gap in disasterinformed educational policy. Participants shared that institutional delays, fragmented communication, and unclear timelines for examinations and curriculum completion were among the factors that drove their decision to leave. These experiences underscore the need to build coordinated policy frameworks that integrate disaster risk reduction, mental health support, and continuity planning into the education sector.

Several participants shared their perceptions of policy gaps and aspirations for resilient structures. They expressed having reshaped their thinking patterns for disaster preparedness based on their experiences, not just for themselves but also for future generations. This sentiment showed a growing awareness among youth regarding the need for systematic preparedness and adaptive measures. Participants advocated for policies that incorporate digital learning platforms, disaster-resilient infrastructure, and organized mental health services within the education sector.

Their narratives show that students see themselves as more than just victims; they see themselves as people who can help make a difference in creating a better response. This finding aligns with contemporary research promoting participatory methodologies in disaster education [68], with youth’s perspectives shaping recovery and preparedness initiatives. Policymakers could not only fill the immediate gaps in education by involving students in planning but also create a culture of resilience and shared responsibility.

This research indicates that the implications of this study have been situational, and cannot be applied globally as prescriptions. In the context of the Pakistani students under discussion, the accounts provided by students suggest that organisational flexibility (flexible assessment schedules and more effective communication during disasters) could reduce extraneous cognitive load and facilitate academic continuity. Such reflections indicate possible directions for a university operating in flood-prone areas to respond to students’ thinking, ensuring that academic activities are adjusted to students’ cognitive realities during disrupted periods.

On a larger scale, the research shows that disaster preparedness in higher education can be improved by considering cognitive load as a key mechanism through which educational disruption can occur. Although the findings cannot be generalised to all disasters, they underscore the importance of context-specific support systems that not only address students’ material recovery but also address the cognitive pressures they face. For policymakers and educators, the research indicates that incorporating student insights into disaster-response plans is worth considering, especially in areas where climate-related disruptions are common.

## Conclusion

The paper offers a qualitative analysis that explores how recent flood disasters have influenced the educational experience of university students in Pakistan. Based on detailed accounts, the study shows how exposure to the disaster reorganises the cognitive requirements of learning by amplifying intrinsic and extraneous load and limiting the likelihood of germane cognitive processing. The study anticipates students’ contextualised experiences in flood-impacted educational contexts rather than attempting to generalise.

The main value of the study is that it provides a theory for applying Cognitive Load Theory to higher education affected by disasters and offers a framework for viewing educational disruption as a cognitive process rather than merely a logistical or psychological consequence. The experience of students in the centre of attention helps reveal the influence of cognitive strain, emotional burden, and structural disruption on the formation of academic engagement, concentration, and progression.

Though the information provided is both context-specific and based on a small sample, it could be included in the body of increasing qualitative research on the usefulness of narrative-based evidence in disaster research. Future research can build on this study by considering different institutional environments, cross-disaster contexts, or recovery patterns. Within the qualitative range of the study, it contributes to understanding the cognition and education experienced by university students in flood-prone areas in response to climate-related disruptions.

## Data Availability

The data supporting the findings of this study are not made publicly available upon publication due to the presence of sensitive personal information and confidentiality agreements established with participants prior to the research. However, the data may be made available upon reasonable request to the authors, provided that the requester meets the necessary ethical and legal requirements and agrees to maintain confidentiality where applicable.
